# E-Cigarette Dependence and Weight-Related Attitudes/Behaviors Associated With Eating Disorders in Adolescent Girls

**DOI:** 10.3389/fpsyt.2021.713094

**Published:** 2021-08-30

**Authors:** Aamina Naveed, Nhan Dang, Pierina Gonzalez, So Hee Choi, Amanda Mathew, Margaret Wardle, Lorra Garey, Ajna Hamidovic

**Affiliations:** ^1^Department of Pharmacy, University of Illinois at Chicago, Chicago, IL, United States; ^2^Department of Public Health, University of Illinois at Chicago, Chicago, IL, United States; ^3^Department of Preventive Medicine, Rush University, Chicago, IL, United States; ^4^Department of Psychology, University of Illinois at Chicago, Chicago, IL, United States; ^5^Department of Psychology, University of Houston, Houston, TX, United States

**Keywords:** E-cigarettes, ENDS, weight preoccupation, body concerns, binge eating, compensatory behavior

## Abstract

**Background:** Although numerous motivations for vaping have been identified in adolescents, no study to date has examined a possible link between vaping and attitudes/behaviors that are associated with eating disorders in adolescent females. Examining this question in adolescent females is especially relevant given the higher prevalence of eating disorders in adolescent girls and women compared to adolescent boys and men.

**Methods:** We recruited 299 girls (between 13 to 17 years old) via Facebook advertisement to complete a REDCap survey, which included the Electronic Cigarette Dependence Index (ECDI), Minnesota Eating Behavior Survey (MEBS), and demographic questions. Data were analyzed using nonparametric Spearman rank correlation test in R.

**Results:** Electronic Cigarette Dependence Index (ECDI) scores were correlated with weight preoccupation (WP), binge eating (BE) and compensatory behavior (CB), but not body dissatisfaction (BD). The following were the results of Spearman correlation tests: (1) WP: rho = 0.13, *p* = 0.02; (2) BD: rho = 0.06, *p* = 0.28; (3) BE: rho = 0.15, *p* = 0.0095; (4) CB: rho = 0.021, *p* = 0.00027.

**Conclusion:** The present study adds to the current literature examining motivations for e-cigarette use in adolescent girls. As eating disorders and e-cigarette dependence are significant public health concerns, our results highlight the need for intervention development.

## Introduction

There has been a rapid increase in the use of electronic nicotine delivery systems (ENDS), including electronic cigarettes (e-cigarettes), among adolescents in recent years. During the period from 2011–2018, US data showed that e-cigarette usage increased from 1.5 to 20.8% in high school students and from 0.6 to 4.9% in middle school students (1). Although this trend was attenuated somewhat from 2019 to 2020, adolescent vaping remains highly prevalent, with 22% of teenagers vaping nicotine in the past 30 days in 2020 (2).

The significant increase in e-cigarettes poses a major public health concern. Adolescents and young adults are the most vulnerable populations to use e-cigarettes (3). E-cigarettes often contain nicotine, which can cause addiction, harm the developing adolescent brain, and may increase the risk for initiating conventional cigarette use (4). Negative health consequences may be more pronounced among adolescents given the opportunity for prolonged use (5). Moreover, since e-cigarette aerosols are a source of exposure to toxic metals (6, 7), they can trigger oxidative stress (8–10).

Given the significant health concerns, it is important to understand motivations and perceptions underlying e-cigarette usage in adolescents. Findings show that curiosity, peer pressure, appealing flavors, and sensation-seeking are all influences for e-cigarette usage among adolescents (11, 12). Additionally, adolescents use e-cigarettes for weight control, with striking sex differences. According to the results of the 2015 Youth Risk Behavior Surveillance survey (13), girls use e-cigarette to lose weight, while boys use them to gain weight.

To date, there have been no findings describing an association between e-cigarette use and attitudes and behaviors associated with eating disorders in female adolescents. Studying this question specifically in females is important because, depending on the psychopathology, data from the National Comorbidity Survey Replication shows two to five time higher prevalence of eating disorders in women compared to men, with the mean age of onset between 18–21 years of age (14, 15). Moreover, smoking cigarettes, as one form of nicotine intake, to control appetite and weight gain is more common among adolescent girls than boys (16–18). As nicotine vaping practices are higher in adults with eating disorders compared to controls (19), we hypothesized a positive correlation between e-cigarette dependence and behavioral/attitudinal symptoms associated with eating disorders in female adolescents (primary analysis). Our secondary analyses include evaluation of e-cigarette dependence and body mass index (BMI). Finally, we characterized changes in e-cigarette use patterns since the COVID-19 outbreak.

## Methods

### Participants

The present study was comprised of 1,161 female adolescents who expressed interest in participating in a study evaluating current e-cigarette use and weight-related concepts. Study participants were between the ages of 13 and 17, current e-cigarette users (defined as using an e-cigarette at least once in the past 30 days and on at least 10 occasions in their lifetime). They were recruited via advertisements posted on Facebook. Exclusion criteria were: (1) non-English speaker and (2) residence outside of the United States.

### Procedures

The survey was conducted from January 17^th^, 2021 to April 18^th^, 2021. Interested individuals were first screened for eligibility, and were then directed to the consent form which explained that the 7-minute online survey is about e-cigarette use, preferences, perceptions, and intentions. They were informed that participating in the online survey may have no more risk than providing information online for other purposes based on the content of the survey. No monetary compensation was offered for study participation. The eligibility questions were the following: (1) “What is your sex? (Male/Female), (2) “How old are you?”, (3) “Do you live in the United States?” (Yes/No), and (4) “In your lifetime, have you used an e-cigarette on more than 10 occasions? (one “occasion” consists of around 15 puffs or lasts around 10 minutes)” (Yes/No). To proceed with the study, interested participants needed to select that they were female, between the ages 13 and 17 and answered “Yes” to the reamining two questions. As the final step prior to taking the survey, study participants filled out a CAPTCHA form. After survey completion, participants were directed to an infographic on the e-cigarette usage, statistics, and consequences of e-cigarette usage. The study protocol was approved by the Institutional Review Board at the University of Illinois at Chicago.

### Measures

*Demographics Questionnaire*. Participants reported sex (Male = 1, Female = 2), race, ethnicity, school status (1 = < than 8^th^ grade, 2 = 8^th^ grade, 3 = 9^th^ grade, 4 = 10^th^ grade, 5 = 11^th^ grade, 6 = 12^th^ grade, 7 = > than 12^th^ grade, 8 = Dropped out of school), height, and weight.

*Electronic Cigarette Dependence Index (ECDI)*. Electronic Cigarette Dependence Index (PSECDI) is a 10 item self-report questionnaire that assesses e-cigarette dependence (20). Participants were asked to report dependence-related concepts, such as the strength of urges to use e-cigarettes, awakening and nightly use, use frequency, cravings/urges, and difficulty quitting. Previous work supports the total score as a valid and reliable index of e-cigarette dependence (20). Scores range between 0 and 20, with 0–3 = not dependent, 4–8 low dependence, 9–12 medium dependence, 13+ = high dependence. The questionnaire and its scoring is included in Supplementary Table 1.

*Minnesota Eating Behavior Survey (MEBS)*. The Minnesota Eating Behavior Survey (MEBS) is a 30-item true/false self-report questionnaire that was developed for the Minnesota Twin Family Study as a measure that allows assessment of a range of behaviors and attitudes associated with eating disorders in individuals of varying ages, including adolescents. It provides quantitative indices of behavioral and attitudinal symptoms associated with anorexia nervosa, bulimia nervosa, and binge eating disorder. The survey is brief, employs a simple response format easily understood by those with limited exposure to psychological questionnaires, and uses a relatively simple language. A brief description of the development and factor analysis of the MEBS was previously presented (21). The total score on the MEBS is an overall measure of disordered eating composed of the following four subscales: Weight Preoccupation (i.e., intense concern with weight and dieting), Body Dissatisfaction (i.e., dissatisfaction with one's body shape and size), Binge Eating (i.e., thoughts about overeating or the tendency to binge eat), and Compensatory Behavior (i.e., the use of compensatory behaviors such as self-induced vomiting or diuretics for weight loss). The questionnaire and its scoring is included in Supplementary Table 2.

*E-Cigarette and Combustible Cigarette Use Patterns*. Our survey asked the following questions to assess cigarette use: (1) Which best describes your current use of CIGARETTES? (Only E-CIGARETTE user/ Both E-CIGARETTE and COMBUSTIBLE CIGARETTES user), (2) Do you smoke COMBUSTIBLE CIGARETTES EVERY DAY? (Y/N), (3) How old were you when you first started using e-cigarettes?, (4) Do you NOW use an e-cigarette EVERY DAY? (Y/N), (5) Does your e-cigarette contain nicotine? (Y/N/I don't know), (6) How have your e-cigarette habits changed since the COVID-19 virus outbreak? (No change/Vaping a bit more/Vaping a lot more/Vaping a little less/Vaping a lot less), and (7) (only for participants indicating lower use of e-cigarettes since COVID-19 outbreak), Why have you been vaping less? The COVID-19 questions were added to qualitatively estimate whether the present study findings are applicable across time.

### Data Analysis

We performed all analyses using the statistical software R. First, we conducted comprehensive exploratory data analyses to identify and correct potential data entry errors, or other inconsistencies. Demographic characteristics of the four ECDI groups (no dependence, low dependence, medium dependence, high dependence) were compared using the chi square test, with post-*hoc* analysis of standardized residuals above 2 indicating statistical significance. We also tested the normality of continuous variables using the “shapiro.test” function in R. All scales of MEBS were not normally distributed and attempts to correct the distribution using conventional (log, sqrt, etc) methods as well as Box-Cox transformation failed. We used the nonparametric Spearman rank correlation test to test the significance of relationships between subscales of MEBS and ECDI, and BMI and ECDI. We considered a p value of 0.05 to be statistically significant.

## Results

### Participant Demographics and Current Cigarette Use

The number of participants completing each step of the survey is the following: (1) pre-screening survey (*n* = 1161), (2) informed consent (*n* = 397), (3) CAPTCHA (*n* = 373), (4) e-cigarette survey (*n* = 299), and (5) educational materials (*n* = 276). As shown in Table 1, the majority of study participants were non-Hispanic White. Results of our demographic analysis show that 91.68% of participants were in high school. Approximately 2/3rds of the participants were daily e-cigarette users, with 15.19% reporting dual e-cigarette and combustible cigarette use. The mean and standard deviation of ECDI in the sample was 10.78 (5.10). Majority of study participants (96.31%) reported that their e-cigarettes contain nicotine, with 3.20% reporting no nicotine, and 0.67% being not sure. The ethnic, racial and school status distributions for the four ECDI groups (no dependence, low dependence, medium dependence, high dependence) were not statisticlly different (Table 1). Daily users were more dependent (medium and high ECDI) than non non-daily users. Dual users had higher e-cigarette dependence (high ECDI) compared to non-daily users.

**Table 1:** Participant demographics according to the electronic cigarette dependence index group.

**Category**	**Options**	**Overall**	**ECDI Category**				***p* value**
			**No Dependence**	**Low Dependence**	**Medium Dependence**	**High Dependence**	
n		299	30	65	79	125	
Race	American Indian or Alaska Native	1 (0.3)	0 (0.0)	0 (0.0)	0 (0.0)	1 (0.8)	0.535
	Asian	5 (1.7)	0 (0.0)	2 (3.1)	1 (1.3)	2 (1.6)	
	Black or African American	4 (1.3)	0 (0.0)	2 (3.1)	0 (0.0)	2 (1.6)	
	Native Hawaiian or Other Pacific Islander	1 (0.3)	0 (0.0)	0 (0.0)	0 (0.0)	1 (0.8)	
	White	245 (81.9)	23 (76.7)	51 (78.5)	67 (84.8)	104 (83.2)	
	More than one race	34 (11.4)	5 (16.7)	9 (13.8)	7 (8.9)	13 (10.4)	
	Unknown	5 (1.7)	0 (0.0)	1 (1.5)	3 (3.8)	1 (0.8)	
	Prefer not to answer	4 (1.3)	2 (6.7)	0 (0.0)	1 (1.3)	1 (0.8)	
Ethnicity	Hispanic	35 (11.8)	2 (6.7)	11 (17.5)	9 (11.5)	13 (10.4)	0.148
	Non-Hispanic	242 (81.8)	25 (83.3)	52 (82.5)	65 (83.3)	100 (80.0)	
	Prefer not to answer	19 (6.4)	3 (10.0)	0 (0.0)	4 (5.1)	12 (9.6)	
School Grade	<8th grade	0 (0.0)	0 (0.0)	0 (0.0)	0 (0.0)	0 (0.0)	0.472
	8th grade	9 (3.0)	1 (3.3)	1 (1.5)	4 (5.1)	3 (2.4)	
	9th grade	42 (14.0)	9 (30.0)	7 (10.8)	10 (12.7)	16 (12.8)	
	10th grade	86 (28.8)	6 (20.0)	23 (35.4)	19 (24.1)	38 (30.4)	
	11th grade	99 (33.1)	10 (33.3)	21 (32.3)	32 (40.5)	36 (28.8)	
	12th grade	47 (15.7)	2 (6.7)	10 (15.4)	12 (15.2)	23 (18.4)	
	> 12th grade	6 (2.0)	1 (3.3)	1 (1.5)	0 (0.0)	4 (3.2)	
	Dropped out of School	10 (3.3)	1 (3.3)	2 (3.1)	2 (2.5)	5 (4.0)	
Daily E-cigarette User	No	94 (31.5)	27 (90.0)	39 (60.0)	15 (19.0)	13 (10.5)	<0.001
	Yes	204 (68.5)	3 (10.0)	26 (40.0)	64 (81.0)	111 (89.5)	
Current Cigarette User	Only E-Cigarette user	218 (72.9)	22 (73.3)	51 (78.5)	65 (82.3)	80 (64.0)	<0.05
	Both E-Cigarette and Combustible Cigarette user	81 (27.1)	8 (26.7)	14 (21.5)	14 (17.7)	45 (36.0)	

### E-Cigarette Dependence and Eating Behaviors/Attitudes

**Figure 1:**
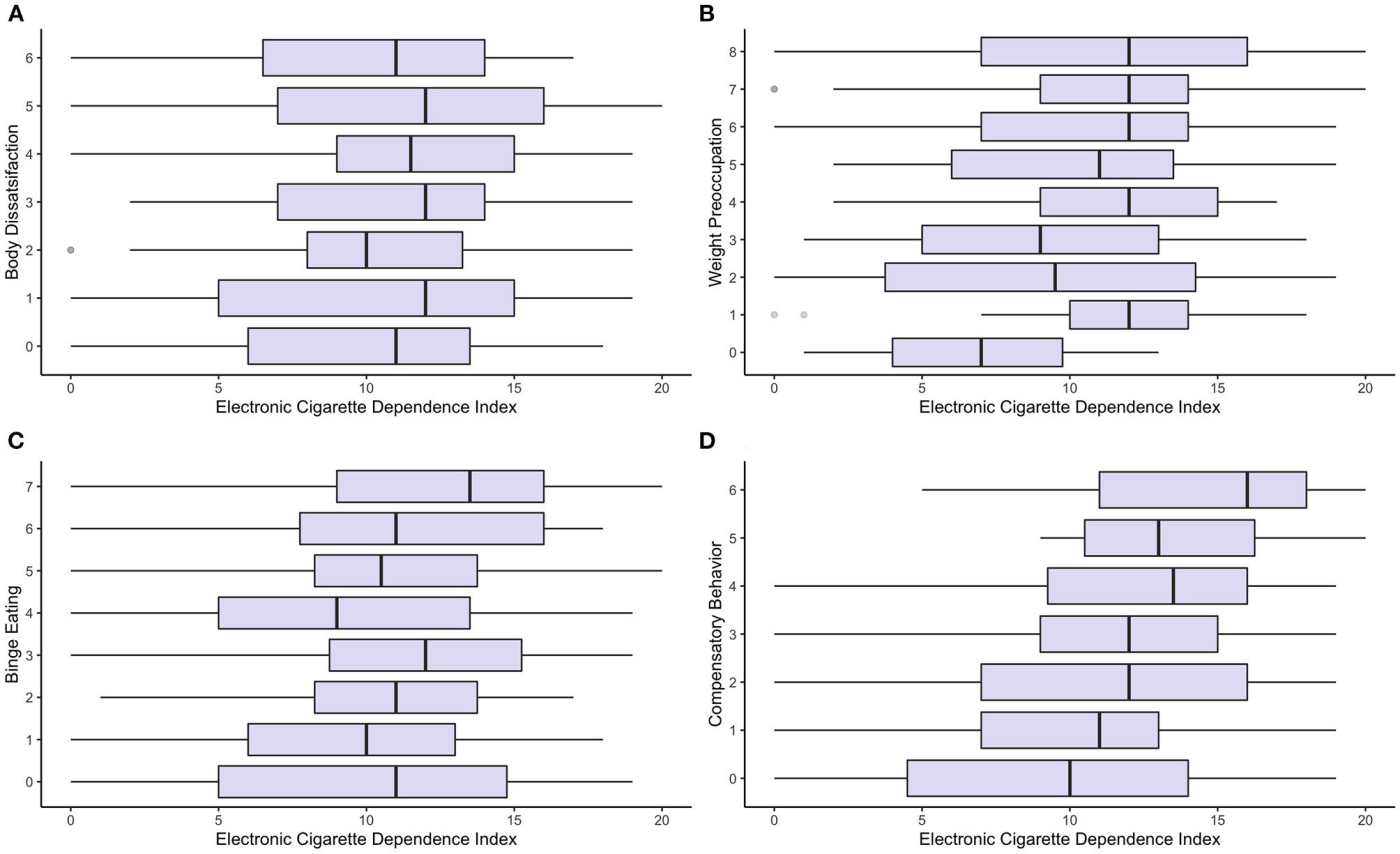
Boxplots of ECDI scores according to MEBS categories. Statistical analyses demonstrated significance between ECDI scores and **(B)** Weight Preoccupation (*p* ≤ 0.05), **(C)** Binge Eating (p ≤ 0.01), and **(D)** Compensatory Behavior (*p* ≤ 0.001). Correlation between ECDI and **(A)** Body Dissatisfaction was not statistically significant.

Electronic Cigarette Dependence Index (ECDI) scores were correlated with three out of four subscales of MEBS: weight preoccupation (WP), binge eating (BE) and compensatory behavior (CB). The following are the results of Spearman correlation tests: (1) WP: rho = 0.13, *p* = 0.02, S = 3853727; (2) BD: rho = 0.06, *p* = 0.28, S = 4175196; (3) BE: rho = 0.15, *p* = 0.0095, S = 3787501; (4) CB: rho = 0.021, *p* = 0.00027; S = 3523183. Figures 1A–D present these findings in graphical form. Table 2 shows means and standard deviations of individual MEBS subscales according to different ECDI levels.

**Table 2:** Electronic cigarette dependence index (ECDI) levels according to minnesota eating behavior survey (MEBS) subscales.

**Minnesota eating behavior survey (MEBS) subscale**	**Electronic cigarette dependence index (ECDI) level**			
	**No Dependence (*n* = 30)**	**Low Dependence (*n* = 65)**	**Medium Dependence (*n* = 79)**	**High Dependence (*n* = 125)**
Weigh preoccupation	4.83 (2.79)	5.38 (2.55)	5.16 (2.56)	5.82 (2.39)
Body dissatisfaction	2.93 (1.98)	3.4 (1.75)	3.14 (1.66)	3.42 (1.74)
Binge eating	3.4 (2.63)	3.46 (2.3)	3.61 (2.21)	4.1 (2.49)
Compensatory behavior	1.27 (1.28)	1.6 (1.43)	1.91 (1.45)	2.18 (1.58)

### Assessment of Association Body Mass Index and E-Cigarette Dependence

Correlation between ECDI and BMI was not significant, with rho = −0.012, *p* = 0.82 and S = 4466919.

### COVID-19 and E-cigarette Use

Majority of study participants reported either no change or higher e-cigarette use since the COVID-19 outbreak. The following is the distribution: (1) no change: 22.82%; (2) vaping a bit more: 29.53%; (3) vaping a lot more: 37.58; (4) vaping a little less: 4.70%; and (5) vaping a lot less: 5.37%. For those answering less vaping, the reason(s) they listed is included in Supplementary Table 3.

## Discussion

The current study demonstrates significant positive correlations between e-cigarette dependency and behavioral/attitudinal symptoms associated with eating disorders in female adolescents. These results suggest that not only is weight loss a significant motive for e-cigarette use in female adolescents; cigarette use is associated with maladaptive attitudes and behaviors toward weight and eating that put individuals at risk for development of eating disorders (22–24). Further, the majority of study participants reported either no change, or more vaping since the COVID-19 outbreak, which suggests that these findings are still applicable even in the context of the pandemic. At the time of the pandemic, adolescents and young adults frequently interacted with friends in social gatherings in some communities (25), where they could obtain e-cigarettes from friends – the most common source among US Youth (12, 26).

Three earlier studies examined weight-related concerns and behaviors to control weight in relation to e-cigarette use among adolescents/young adults, generally suggesting associations consistent with ours. A recent study administered a survey assessing weight concerns (weight and body shape) to 470 college students (65% female) (27). The investigators did not find an association between weight concerns and e-cigarette use status (Lifetime user Y/N). This finding is different from the one in our study possibly due to age (middle/high school vs. college), sex (female vs. both), and e-cigarette assessment (lifetime use [Y/N] vs ECDI). In a second large-scale survey of Chinese middle and high school students, (28) found a significant association between current e-cigarette use and specific unhealthy weight loss behaviors (eating less food, taking laxatives, taking diet pills, and going without eating for 24 hours or more), although there was no association with general self-report of trying to control weight (i.e., “Are you doing something to lose or to keep from gaining weight?”). Similarly, a third large-scale survey of Korean adolescents aged 13–18 found that unhealthy weight control behaviors (i.e., one-food diet, fasting, diet pill use, and purging) were more common among current e-cigarette users vs. ever- and never-users (29).

Body concerns can be operationalized in two domains - weight preoccupation and body dissatisfaction, and we found different results across these two domains. In the present study, only weight preoccupation was associated with e-cigarette dependence. Although the two domains are significantly correlated (r =0.66), the work by (30) showed that, in nonclinical female adolescents, weight and shape preoccupation was more consistently related to current eating disorder symptoms than body dissatisfaction. However, results of several studies show that body concerns predict increased risk for future onset of full or subclinical eating disorders (23, 31, 32). Futher, our results are cross-sectional, so in the present study e-cigarette dependence may either be a result of greater present eating disorder symptomatology, or a leading indicator of future eating disorder symptomatology, which typically peaks in late adolescence (33). The possibility that e-cigarette use is part of significant present symptoms is supported by findings that e-cigarette dependence is also significantly correlated with actual binge eating and compensatory behavior, suggesting presence of current concerning behavior in female adolescents with e-cigarette dependence. However, future longitudinal research is needed to discern whether e-cigarette dependence primarily develops as part of existing disordered eating, or is a leading predictor of future problems.

Several mechanisms may account for the co-occurrence of e-cigarette use and weight preoccupation/disordered eating behavior among adolescent girls. First, adolescents with weight preoccupation may be more likely to use e-cigarettes for appetite control and weight loss motives (34). Second, the co-occurrence of e-cigarette use and eating disorder symptomatology may reflect shared biobehavioral risk factors, such as impulsivity and executive functioning deficits (35). These risk factors may predispose adolescents to both addictive behaviors and psychopathology, and may differ by gender (36). Accordingly, mental health screening may be indicated to facilitate early recognition of disordered eating attitudes/behaviors and other mental health challenges, particularly among adolescent females using e-cigarettes.

The concerns about relationships between e-cigarette use and disordered eating are unlikely to abate in the near future. E-cigarette companies are actively developing e-cigarette technologies related to e-cigarettes and weight loss. As patents can provide insight into the direction of product marketing and design innovations made by the industry, (37) identified a number of patents proposing to add weight loss drugs which alter body metabolism/nutrient absorption or appetite suppressants to e-liquids. These findings are not surprising given the long history of cigarette companies' approach to promote the idea that cigarette smoking is helpful for controlling body weight. For example, cigarette advertisements from the 1930s suggested that women should “reach for a cigarette instead of a sweet” (38). Almost 100 years later, messages related to nicotine use and weight issues are getting through via social media, among other outlets (39, 40).

The current study had limitations. The data collected from participants was based on their ability and willingness to respond accurately. These concerns were mitigated by administering an anonymous survey. Our approach (i.e., describing the study objective as evaluation of weight-related issues and e-cigarette use) possibly selected a sample with more weight concerns/disordered eating than the general population. This is based on the finding that mean (SD) for the 4 measures (WP, BD, BE and CB) are the following for our sample [5.45 (2.52), 3.29 (1.74), 3.75 (2.39), 1.88 (1.50)] *vs*. healthy adolescent girls of similar age [3.61 (2.51); 2.74 (2.24), 1.59 (1.57), 0.52 (0.99)] (41). The findings of the present study would have been strengthened by comparing e-cigarette dependence groups according to the self-reported history of eating disorder diagnoses. This would have required a greater power than we had for the present comparison assessing attitudes/behaviors that are associated with eating disorders; nonetheless, this analysis would have solidified the study findings. Moreover, our database does not contain information about the concentration of nicotine in e-cigarettes.

Preoccupation with body weight and shape, binge eating and compensatory behaviors are associated with serious eating disorders, while e-cigarette use in adolescents can trigger oxidative stress and lead to negative long-term consequences, including addiction. Our study demonstrates association between e-cigarette use and behavioral/attitudinal symptoms associated with eating disorders, highlighting the need to for eating disorder/ e-cigarette dependence interventions in adolescent girls.

## Data Availability Statement

The original contributions presented in the study are included in the article/Supplementary Material, further inquiries can be directed to the corresponding author/s.

## Ethics Statement

The studies involving human participants were reviewed and approved by Institutional Review Board at the University of Illinois at Chicago. Written informed consent for participation was not required for this study in accordance with the national legislation and the institutional requirements.

## Author Contributions

AH designed the study, analyzed study data, and wrote the initial and final manuscript versions. AN wrote, executed the protocol, and resolved regulatory issues. ND analyzed all study data. PG executed the protocol. MW, AM, and LG assisted with regulatory issues and manuscript writing. SC assisted with manuscript submission. All authors contributed to the article and approved the submitted version.

## Conflict of Interest

The authors declare that the research was conducted in the absence of any commercial or financial relationships that could be construed as a potential conflict of interest.

## Publisher's Note

All claims expressed in this article are solely those of the authors and do not necessarily represent those of their affiliated organizations, or those of the publisher, the editors and the reviewers. Any product that may be evaluated in this article, or claim that may be made by its manufacturer, is not guaranteed or endorsed by the publisher.
