# Supplementary material for: E-Cigarette Dependence and Weight-Related Attitudes/Behaviors Associated With Eating Disorders in Adolescent Girls
Source: Front Psychiatry. 2021 Aug 30;12:713094. doi: 10.3389/fpsyt.2021.713094 (PMC8437144; doi:10.3389/fpsyt.2021.713094)
Supplement: Supplementary file 1 [file Table_1.DOCX]

Supplementary Table 1. Electronic Cigarette Dependence Index (ECD) Scoring

| Questions | Scoring |
| --- | --- |
| 1. How many cigarettes [times] per day do you usually smoke [use your electronic cigarette]? ([assume that one “time” consists of around 15 puffs or lasts around 10 minutes]) | 0–4 times/day = 0, 5–9 = 1, 10–14 = 2, 15–19 = 3, 20–29 = 4, 30+ = 5 |
| 2. On days that you can smoke [use your electronic cigarette] freely, how soon after you wake up do you smoke your first cigarette of the day [first use your electronic cigarette]? | 0–5 mins = 5, 6–15 = 4, 16–30 = 3, 31–60 = 2, 61–120 = 1, 121+ = 0 |
| 3. Do you sometimes awaken at night to have a cigarette [use your electronic cigarette]? | Yes = 1, No = 0 |
| 4. If yes, how many nights per week do you typically awaken to smoke [use your electronic cigarette]? | Scoring: 0–1 nights = 0, 2–3 nights = 1, 4+ nights = 2 |
| 5. Do you smoke [use an electronic cigarette] now because it is really hard to quit? | Scoring: Yes = 1, No = 0 |
| 6. Do you ever have strong cravings to smoke [use an electronic cigarette]? | Scoring: Yes = 1, No = 0 |
| 7. Over the past week, how strong have the urges to smoke [use an electronic cigarette] been? | None/Slight = 0, Moderate/Strong= 1, Very Strong/Extremely Strong = 2 |
| 8. Is it hard to keep from smoking [using an electronic cigarette] in places where you are not supposed to? | Yes = 1, No = 0 |
| When you haven’t used tobacco [an electronic cigarette] for a while or when you tried to stop smoking [using]… 9. Did you feel more irritable because you couldn’t smoke [use an electronic cigarette]? | Yes = 1, No = 0 |
| 10. Did you feel nervous, restless, or anxious because you couldn’t smoke [use an electronic cigarette]? | Yes = 1, No = 0 |
