# Supplementary material for: E-Cigarette Dependence and Weight-Related Attitudes/Behaviors Associated With Eating Disorders in Adolescent Girls
Source: Front Psychiatry. 2021 Aug 30;12:713094. doi: 10.3389/fpsyt.2021.713094 (PMC8437144; doi:10.3389/fpsyt.2021.713094)
Supplement: Supplementary file 2 [file Table_2.DOCX]

| Supplementary Table 2. Minnesota Eating Behavior Survey |
| --- |
| I can eat sweets and starches (like potatoes, pasta and bread) without feeling upset or nervous. |
| I often diet to control my weight.  (WP) |
|  |
|  |
| My stomach is too big.  (BD) |
|  |
|  |
| I eat when I'm upset about things.  (BE) |
|  |
|  |
| I have thought about throwing up (vomiting) to lose weight.  (CB) |
|  |
|  |
| Sometimes I stuff myself with food.  (BE) |
|  |
|  |
| I think a lot about dieting (or losing weight).  (WP) |
|  |
|  |
| My thighs are about the right size.  (BD) |
|  |
|  |
| Sometimes I completely stop eating for more than a day to control my weight.  (CB) |
|  |
|  |
| I feel terribly guilty if I overeat.  (WP) |
|  |
|  |
| I am really afraid of gaining weight.  (WP) |
|  |
|  |
| The shape of my body is fine.  (BD) |
|  |
|  |
| Sometimes I use laxatives (like Ex-Lax or Correctol) to control my weight.  (CB) |
|  |
|  |
| My weight is very important to me.  (WP) |
|  |
|  |
| Sometimes I eat lots and lots of food and feel like I can't stop.  (BE) |
|  |
|  |
| I sometimes use diet pills (like Dexatrim, Dietac, or Acutrim) to control my weight.  (CB) |
|  |
|  |
| I'm always wishing I was thinner.  (BD) |
|  |
|  |
| I think a lot about overeating (eating a really large amount of food).  (BE) |
|  |
|  |
| Sometimes I have a hard time telling if I'm hungry or not. |
|  |
|  |
| I exercise to control my weight more than other women my age. |
|  |
|  |
| My hips are just the right size.  (BD) |
|  |
|  |
| Sometimes, when I'm with other people, I won't eat much, but later, when I'm alone, I'll eat a lot.  (BE) |
|  |
|  |
| I feel fat or stuffed even after eating a normal meal.  (WP) |
|  |
|  |
| If I gain a pound, I worry that I will keep gaining more and more weight.  (WP) |
|  |
|  |
| Sometimes I make myself throw up (vomit) to control my weight.  (CB) |
|  |
|  |
| Sometimes I eat by myself so that others won't know what I'm eating.  (BE) |
|  |
|  |
| When I get upset, I'm afraid that I will start eating.  (BE) |
|  |
|  |
| I often weigh myself to see if I am gaining weight.  (WP) |
|  |
|  |
| I sometimes use medicine that makes me lose water (diuretics like Sunril, Aqua-Ban, Pamprin, or Midol PMS) to control my weight.  (CB) |
| WP: Weight Preoccupation subscale; BD: Body Dissatisfaction  subscale; BE: Binge Eating subscale; CB: Compensatory Behavior subscale |

To score the MEBS, each item answered in the symptomatic or pathological direction (usually yes) receives 1 point and receives no point if the item was scored in the non-pathological direction (usually no). Four items require reverse scoring to enable interpretation of the total score and subscale scores as indices of pathological eating attitudes and behaviors. MEBS total scores comprise the sum of scores from all 30 items. Each of the four subscale scores is obtained by summing the scores of all items that

comprise the subscale. Further information can be found in von Ranson et al (2005).
