# Supplementary material for: E-Cigarette Dependence and Weight-Related Attitudes/Behaviors Associated With Eating Disorders in Adolescent Girls
Source: Front Psychiatry. 2021 Aug 30;12:713094. doi: 10.3389/fpsyt.2021.713094 (PMC8437144; doi:10.3389/fpsyt.2021.713094)
Supplement: Supplementary file 3 [file Table_3.DOCX]

| Supplementary Table 3. Reasons for Vaping Less Since COVID-19 Outbreak |
| --- |
| **HEALTH REASONS** |
| Because vaping is unhealthy |
| Covid attacks the lungs and vaping with the chance of getting covid isn't appeasing thing |
| I worried if I got covid if I would die |
| Worried about getting sick |
| I now get bronchitis 4 times a year |
| I don't want to compromise my lungs |
| **ACCESSIBILITY REASONS** |
| Access issue |
| Harder to get |
| Harder to get a new one |
| I don't have as much access to vapes |
| I don’t have access to a vape |
| Less access |
| Less available |
| Not easily accessible |
| **CESSATION REASONS** |
| Just attempting to ween myself off/ use less |
| Just didn’t want to do it |
| Prior to the pandemic decided I'd only make vaping a social thing to ween off nicotine |
| Think it’s stupid and I should quit |
| Wanted to quit |
| **FINANCIAL REASONS** |
| No money |
| No money to buy anything |
| I only buy nicotine when I have extra money |
| **SOCIAL REASONSE** |
| I'm at home more |
| No access to people with e-cigs |
| I am not in party settings where I use it the most |
| I got accustomed to going to the bathroom at school every period to use it, now I can go hours without using it |
| **OTHER** |
| Have to wear a mask and can't just vape in public |
| I smoke marijuana instead |
| Plug can't meet as often |
